# Patellar Clunk Syndrome Following Posterior Stabilized Total Knee Replacement: Report of Two Cases

**DOI:** 10.7759/cureus.11435

**Published:** 2020-11-11

**Authors:** Ali H Chamseddine, Ibrahim Haidar, Mohammad Jawad Rahal, Ali Asfour, Mohammad O Boushnak

**Affiliations:** 1 Orthopedic Surgery, Sahel General Hospital, Lebanese University, Faculty of Medical Sciences, Beirut, LBN; 2 Orthopedic Surgery, Lebanese University, Faculty of Medical Sciences, Beirut, LBN

**Keywords:** patellar clunk, patellar crepitation, total knee replacement, posterior stabilized total knee replacement, total knee replacement complications

## Abstract

Patellar clunk syndrome (PCS) occasionally occurs after posterior stabilized total knee replacement (PS-TKR), and is characterized by a painful palpable audible clunk of the patella when the knee moves from flexion to extension. It has been classically attributed to the formation of fibrous nodule at the junction of the proximal pole of the patella and the undersurface of the distal quadriceps tendon. However, various intra-articular peripatellar proliferative fibrous formations have also been reported with a wide spectrum of symptoms, ranging from crepitation to frank patellar clunk. Treatment of the syndrome remains essentially surgical, and usually consists of resection of the fibrous nodules. This paper reports two cases of PCS and aims at bringing attention to this entity in terms of pathogenesis, clinical diagnosis, and treatment, through a review of the literature.

## Introduction

Patellar clunk syndrome (PCS) is a patellofemoral dysfunction that may occur in up to 18% of patients with posterior stabilized total knee prosthesis (PS-TKR) [1], usually during the first year following total knee replacement [2-4]. It consists of painful palpable and audible clunk, catch, grinding, or jumping of the patella when the knee moves from flexion to extension, and is commonly related to the formation of fibrous nodule at the junction of the proximal pole of the patella and the undersurface of the distal quadriceps tendon [5].

We report herein two cases of PCS. Our aim is to bring attention to this entity in terms of pathogenesis, clinical diagnosis, and treatment, along with a review of the literature.

## Case presentation

Case 1

**Figure 1:**
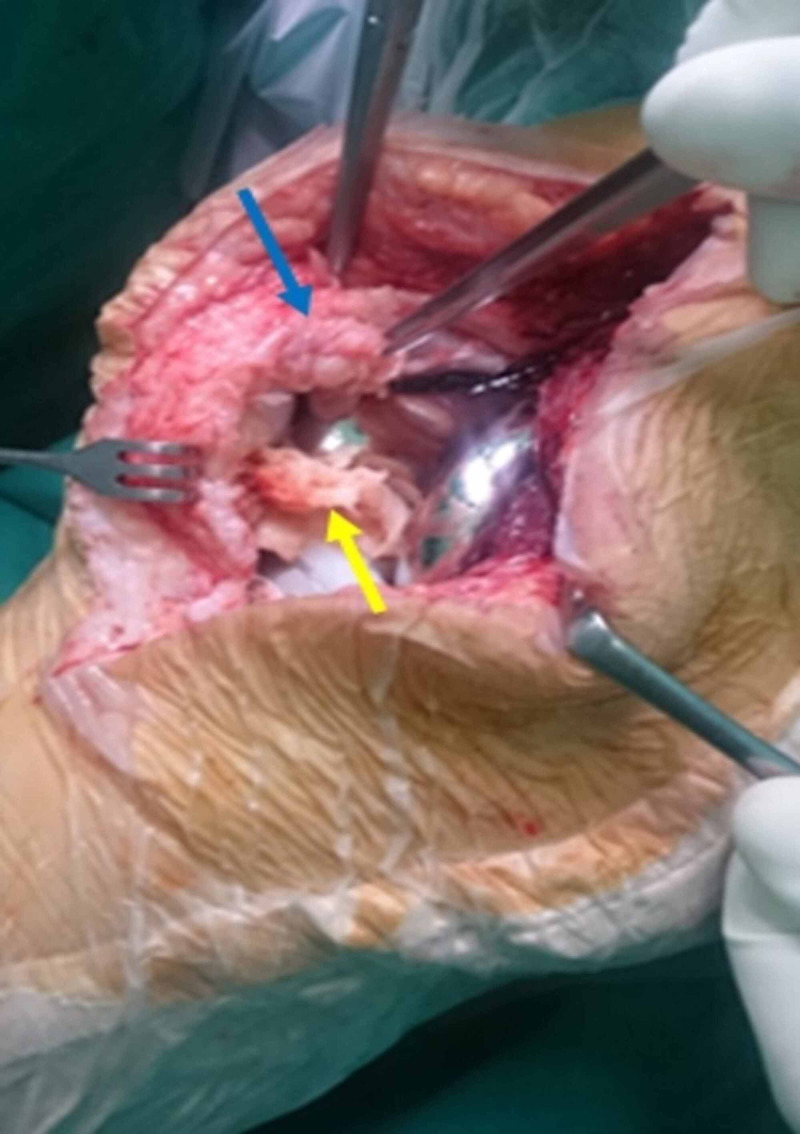
Intraoperative view of Case 1 showing two nodules of considerable size developed at the proximal and distal poles of the patellar button (blue and yellow arrows, respectively) Note that the proximal nodule (blue arrow) obliterates the suprapatellar space and overlaps the proximal patellar button, and the distal nodule (yellow arrow) completely occupies the intercondylar box of the femoral component.

**Figure 2:**
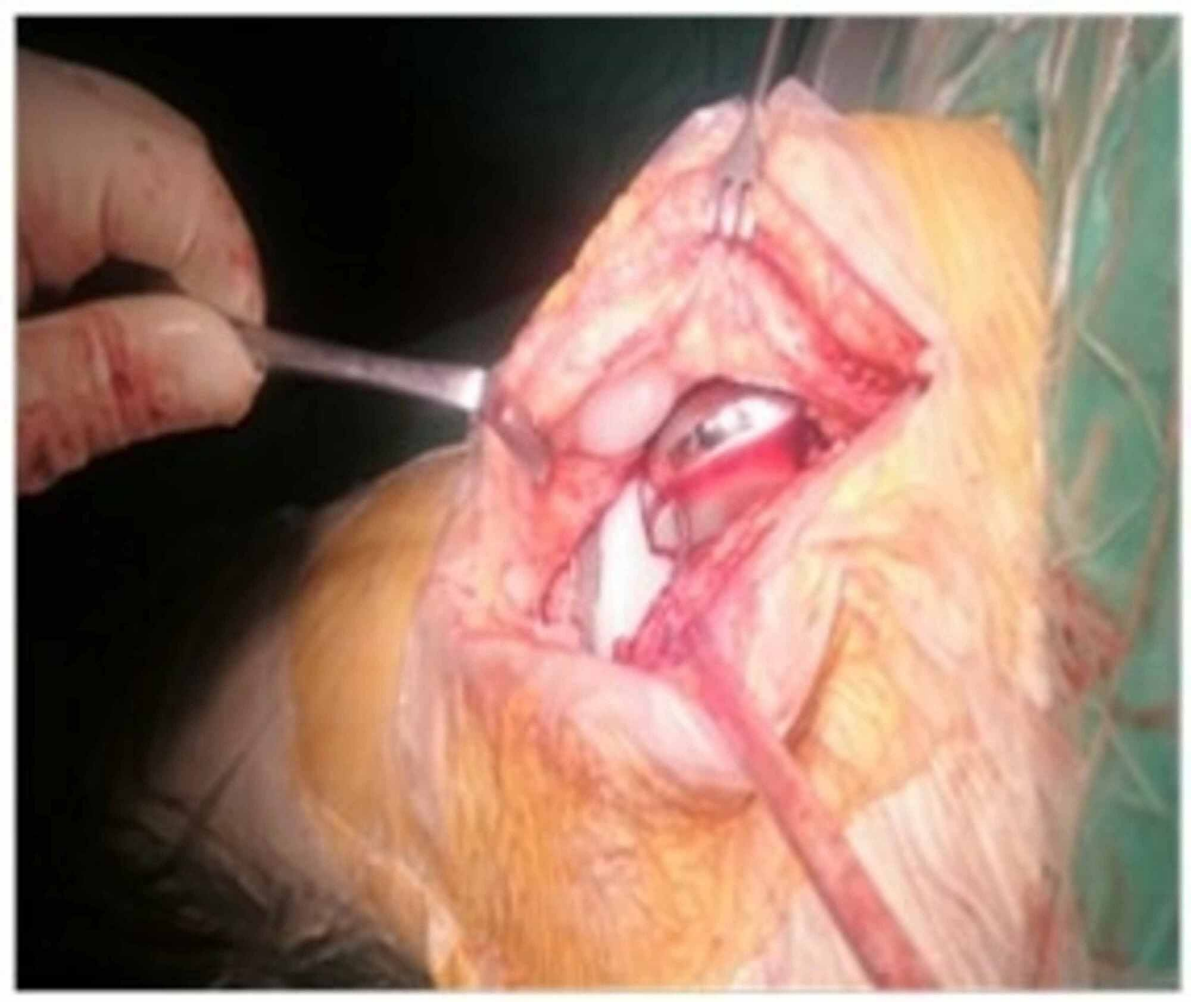
Intraoperative view of Case 1 after debridement The prosthetic joint has been adequately debrided and is now clear from any fibrous formation.

**Figure 3:**
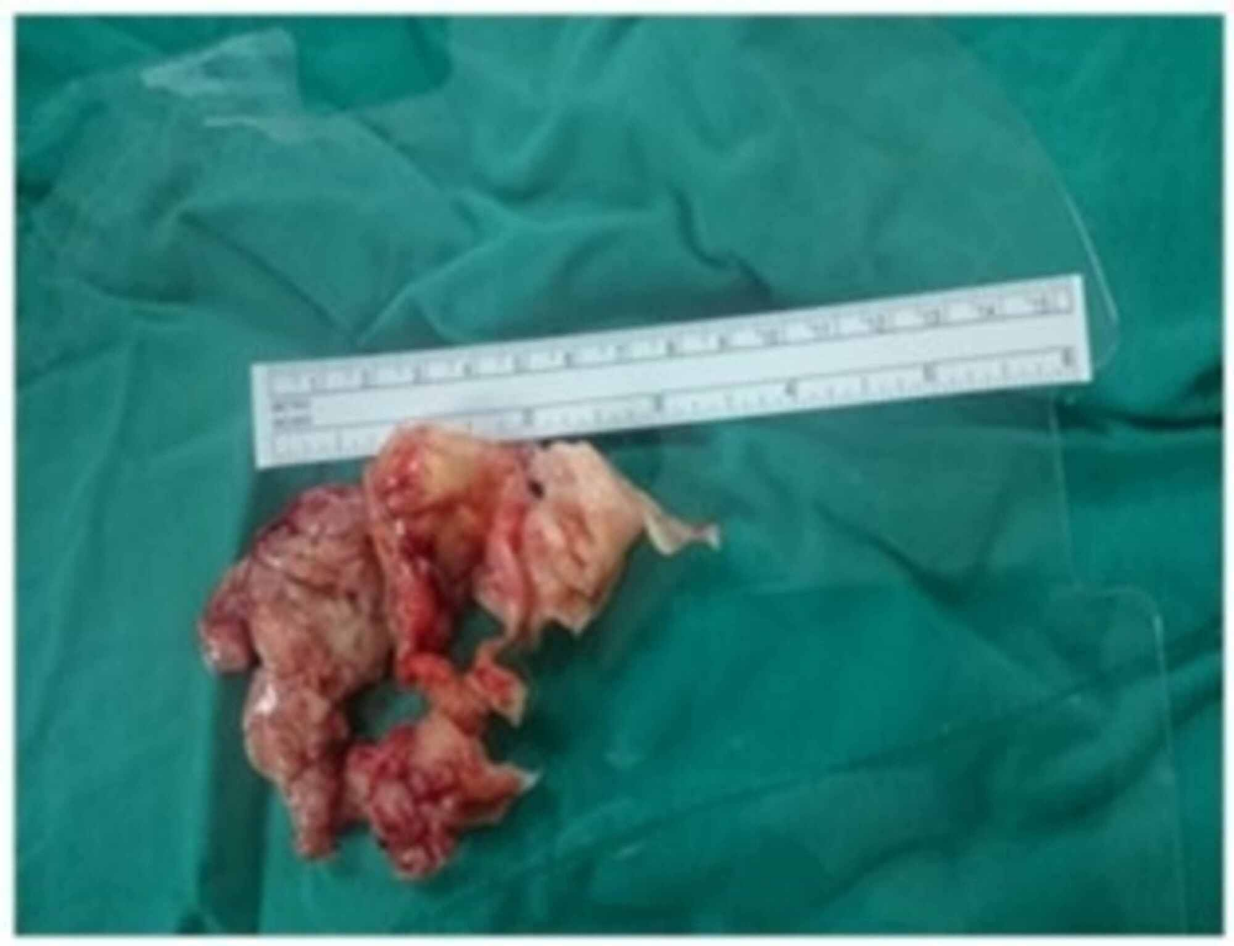
Appearance of the fibrous formation after removal in Case 1

A 65-year-old female was operated with left total knee replacement for painful disabling knee osteoarthritis, using posterior stabilized fixed bearing PFC Sigma total knee prosthesis. The operative procedure was performed with the use of tourniquet through medial parapatellar arthrotomy, according to standard surgical technique and without difficulties or complications. Synovectomy with removal of the fat pad and meniscal remnants were performed, along with excision of the soft tissue and exuberant bone around the patella, which was replaced with mild medialization. All components were cemented, and the prosthetic knee was intraoperatively stable without patellar maltracking. The wound was closed with intra-articular aspiration drain. Weight-bearing and knee mobilization with muscle strengthening were started after removal of the drain, two days post-surgery. At three months postoperatively, the patient complained of disabling painful left knee "click" while climbing stairs and standing up from chairs. Left knee physical exam demonstrated visible and audible painful "clunk" with "jerk" of the patella when the knee was moved from flexion to extension, at around 30 degrees from full extension, as well as when rising from sitting position from chair. Radiographic assessment showed a good position of the prosthetic components without any alarming signs. A diagnosis of PCS was established based on signs and symptoms, and the patient was operated four months after the index procedure. Repetitive medial parapatellar arthrotomy revealed two separate fibrous formations: one nodule was found at the junction of the proximal pole of the patella, with the undersurface of the distal quadriceps tendon completely obliterating the suprapatellar space, and the second nodule was found at the distal pole of the patella extending into the intercondylar box of the prosthetic femoral component (Figure 1). Careful complete excision of both nodules was performed, and closure was performed following abundant irrigation (Figures 2, 3). The patient underwent a few sessions of postoperative physiotherapy. At two-year follow-up, the knee was asymptomatic with no patellar clunk recurrence.

Case 2

**Figure 4:**
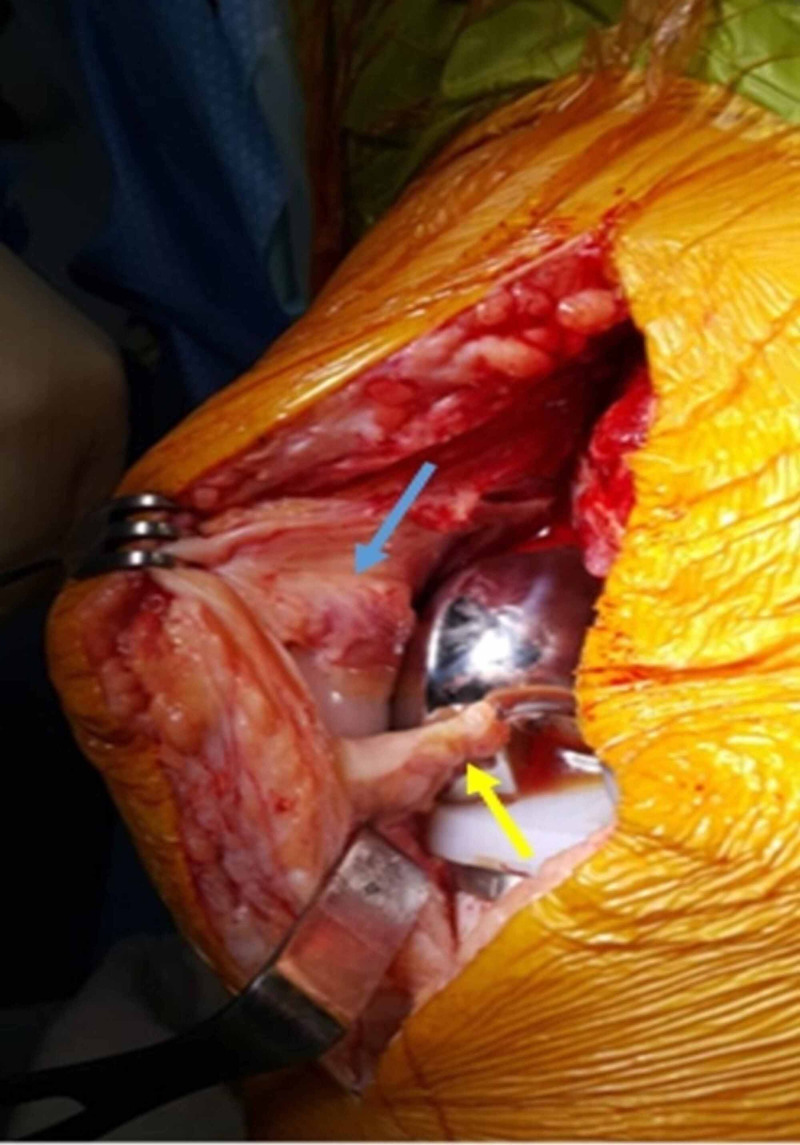
Intraoperative view of Case 2 showing the overlap of the proximal nodule (blue arrow) with the proximal pole of the patellar button, and the development of the distal nodule (yellow arrow) into the intercondylar box of the femoral component

**Figure 5:**
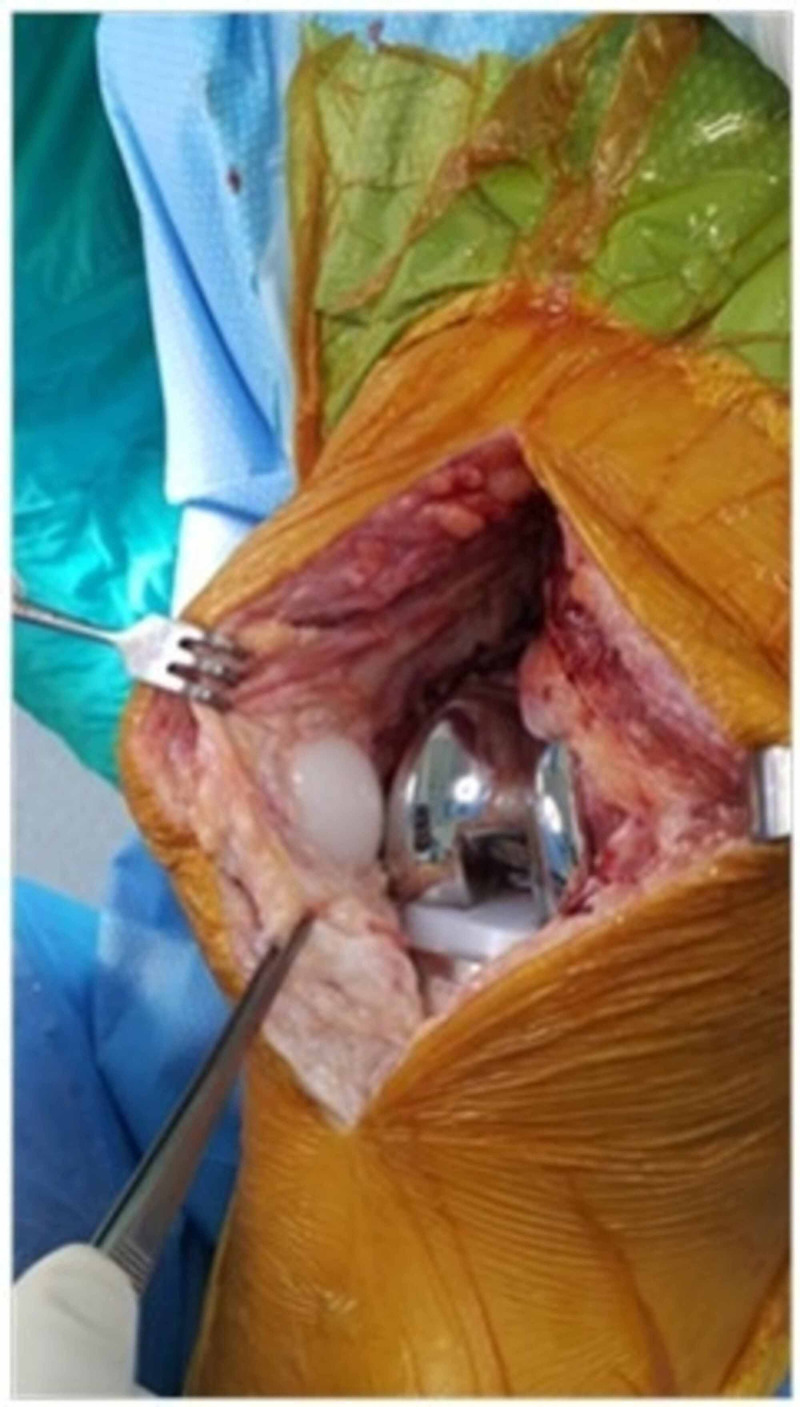
Intraoperative view of Case 2 after complete resection of the nodules The patellar button and the intercondylar box of the femoral component are now clear and well visualized.

A 66-year-old female presented five months after right total knee replacement performed as described above, with the same complaint of disabling right knee painful "click" while climbing stairs and rising from chairs. Physical exam findings were similar to those of the previous patient, with no abnormal findings on radiographic control. The patient was reoperated six months after the index procedure, with repetitive medial parapatellar arthrotomy that showed two fibrous nodules very comparable to the previous case: the first nodule, developed at the undersurface of the distal quadriceps tendon, was covering the proximal pole of the patellar button, while the second nodule, developed at the articular aspect of the junction of the distal pole of the patella with the patellar tendon, was erected into the intercondylar notch of the femoral component (Figure 4). The nodules were excised and the wound was closed after irrigation (Figure 5). At one-year follow-up, the patient's right knee was symptom-free, with no signs of patellar clunk recurrence.

## Discussion

PCS usually occurs between three and 12 months - occasionally up to 34 months - after PS-TKR with patellar resurfacing and rarely, after patellar non-resurfacing procedure [2]. A wide spectrum of patellar-related symptoms, ranging from crepitation to fully developed PCS, has also been depicted after PS-TKR [6]. In 1989, Hozack et al. [5] first coined the term "Patellar Clunk Syndrome" after reporting on three cases of patellofemoral pain, with sensation of patellar "catch" or "clunk" following PS-TKR in three patients. These authors [5] attributed the symptoms to a prominent fibrous nodule formation at the junction of the proximal patellar pole and the articular aspect of the quadriceps tendon, essentially secondary to the impingement of the undersurface of the tendon into the sharp anterosuperior edge of the intercondylar notch of the femoral component. The nodule was found to be entrapped into the intercondylar box of the femoral component during flexion, and suddenly dislodged when the knee moves in extension to 30-45 degrees of full extension, resulting in a painful audible clunk [5]. Although several authors reported up to 12%-21% of patellofemoral early complications with the original Insall-Burstein knee prosthesis (IB I) released in 1978 [7], the true incidence of the syndrome was probably underestimated due to lack of knowledge at that time.

Several modifications were consequently made to the original prosthetic design, in an attempt to lessen the incidence of these complications. In the second-generation modular Insall-Burstein II Posterior Stabilized design (IB II PS), a more rounded sagittal curvature of the femoral condyle, along with a deepened trochlear groove, contributed to the decrease of PCS incidence to 2%-5% [2,3,5,7,8]. In the third-generation prosthesis, the NexGen Legacy Posterior Stabilized (NG LPS), additional significant modifications were introduced in order to improve patellofemoral mechanics, and thereby reduce patellofemoral complications, including PCS [9]: increased number of femoral component sizes for more exact size matching and less overstuffing, side-specific femoral components to allocate 7° oblique orientation of the patellar groove, posterior shift of the cam-post mechanism with consequent trochlear deepening, and finally lengthening of the trochlea to allow progressive and smoother transition with the distal femoral condyles [9]. Ip et al. [3] compared the incidence of PCS between two series of consecutive patients who underwent PS-TKR with two different models; they found 7.5% PCS in 80 knees with IB II PS, and 0% in 50 knees with NG LPS. Similarly, Clarke et al. [9] noted 0% PCS at a mean 48-month follow-up in a series of consecutive 238 knees that received NG LPS prosthesis; however, 36% of these knees underwent primary release of the lateral patellar retinaculum. At two-year (minimum) follow-up, Maloney et al. [10] found a 3.9% incidence of PCS in a group of 179 knees with IB II PS, compared to 0% in another group of 210 knees with the Advance PS knee prosthesis. As the patellar groove of the Advance PS implant was posterodistally extended by 7.5 mm compared to the IB II PS implant, the authors stated that this design change essentially eliminated the problem of patellar clunk, by increasing the flexion angle needed for the proximal pole of the patella to fall into the intercondylar notch.

A comparative study of three cohorts of patients consisting of 80 IB II PS, 60 Anatomic Modular Posterior Stabilized Knee (AMK PS), and 106 Low Contact Stress Rotating Platform (LCS) total knee replacements, showed a PCS incidence of 8.8%, 3.3%, and 0%, respectively [11]. A shallow configuration and absence of lateral build-up of the patellar groove, such as in the IB II PS, were incriminated in the occurrence of patellar problems; a deeper and more distally extended groove, along with a narrower and more posteriorly located intercondylar box, such as in the femoral components of the AMK, LCS, and NG designs, were deemed to contribute to a decreased PCS incidence [3,11]. Ip et al. [11] stated that the absence of distinct intercondylar box in the LCS design does not allow soft-tissue entrapment into the intercondylar region of the femoral component, and therefore partly contributed to the absence of PCS with this type of prosthesis. In 2009, Fukunaga et al. [4] defined the "intercondylar box ratio" as the ratio of the length of the intercondylar box to the anteroposterior size of the femoral component. They noted a high incidence (3.3% to 18.3%) of PCS in the literature when the ratio was > 0.7. The femoral component of the IB II PS prosthesis has an intercondylar box ratio (ICBR) of 0.71-0.72; its use was associated with 7.5% and 10.2% PCS in the reports by Ip et al. [3] and Yau et al. [6], respectively. An ICBR of 0.84-0.85, such as in the AMK PS prosthesis, was correlated with PCS by 3.3% [11] and 18.3% [6] in two different publications. Incidences of 12% and 13.3% were, respectively, encountered in fixed bearing and in mobile-bearing PFC Sigma prosthesis [4]; both types have an ICBR of 0.85-0.87. In contrast, prosthetic designs with ICBR of 0.56-0.59 (NG LPS) and 0.62 (Advanced Posterior Stabilized) were associated with a 0% incidence of the syndrome [3,9,10]. Snir et al. [12] also reported a high incidence (11.7%) of PCS with the mobile-bearing PFC Sigma. However, Fukunaga et al. [4] stated that this might be partly related to the 3° external rotation surgically set to the femoral condyles according to the standard surgical technique, and found a correlation between the incidence of the PCS and the postoperative patellar tilt. Frye et al. [13] speculated that the modifications introduced to the conventional PFC Sigma posterior stabilized implant, such as a deeper trochlear groove with smoother transition of the intercondylar box, completely eliminated the problem of PCS; they stated that the modified prosthetic design better accommodates the synovial nodule, rather than reducing its development [13].

Certain authors [8,14] considered that the etiology of patellar clunk and crepitus is multifactorial and most likely involves the prosthetic design, surgical trauma, components position, alteration of the joint line, patellar height, and patellar button-patellar bone composite thickness. Based on previously defined measurements and parameters, Yau et al. [6] stated that the occurrence of PCS is significantly related to the postoperative Insall-Salvati ratio, the position of the proximal pole of the patella from the distal end of the femoral prosthesis [14], the postoperative patellar button height [14], the relative position of the tibial tray with reference to the patellar tendon [6], and the congruency of the patellar button defined by the tilt of the patella, and its lateral displacement from the midline of the trochlear groove of the femoral component on the skyline view [6] they did not find a significant link between PCS and the change in position of the joint line with reference to tibial tuberosity [6]. In a retrospective multicenter case-control study, Dennis et al. [15] identified 6 factors that statistically amplified the risk of patellar clunk or crepitus following PS-TKR: reduced preoperative or postoperative patellar tendon length, thin postoperative composite patellar component thickness, reduced patellar component size, increased posterior femoral condylar offset, and history of previous knee surgery. Nevertheless, others believe that the geometry of the intercondylar box is the single most important factor in the development of PCS [3,9,10,12,13,15]. Various intra-articular peripatellar proliferative fibrous formations may contribute to the occurrence of a wide spectrum of symptoms following PS-TKR, ranging from crepitation to frank patellar clunk syndrome [6]. They include impinging nodule at the proximal patellar pole [2,5], impinging nodule in the intercondylar notch [11], impinging hypertrophic synovitis between the femoral and tibial components [8], impinging posterior cruciate ligament stump [16] and arthrofibrosis [17].

Consequently, several classifications have been made. Takahashi et al. [18] suggested a classification into three types: type I corresponds to fibrous nodule at the proximal pole of the patellar button and represents the patellar clunk syndrome, type II is an impinging hypertrophic synovitis, and type III is a combination of the previous two types. Carro and Suarez [19] reported a thickened fibrotic nodule in the intercondylar notch, impinging with the polyethylene button, and proposed a classification into two types: type A is intra-articular complex fibrous bundle reactions, and type B is intra-articular fibrous nodules that may exist in the patellofemoral area and the intercondylar notch. Despite the multifactorial etiologic character of the syndrome, preventive measures have been recommended at the time of index surgery: excise any hypertrophic fibro-synovial tissues at the junction of the undersurface of the distal quadriceps tendon with the proximal border of the patella [1], use prosthetic design with low ICBR [4], avoid insertion of downsized femoral component in flexed position [14,15], avoid anterior placement of the tibial tray [6], avoid any contact of unresurfaced patella with the femoral prosthetic component, by placing the patellar button as proximal as possible, but without overhanging or extending it beyond the superior border of the bony patella [1,15], avoid over-resection of the patella, and avoid insertion of small-thin patellar button that induces a thinner patellar composite thickness [1,15]. Finally, Conrad et al. [1] stated that patients with a history of previous knee surgery, or with decreased preoperative patellar tendon length, should be informed about the increased risk of developing PCS.

Treatment of the established syndrome consists of non-operative measures, including physiotherapy and injections, and operative measures corresponding to either arthroscopic or open excision of the nodule. Quadriceps exercises for a few months, surprisingly, may show improvement over time, for uncertain reasons, in 20 to 60% of patients [2,10,11,12]. Ip et al. [11] recommend arthroscopic treatment after failure of conservative measures, and open treatment for patients in whom it is predicted to perform component revision and/or soft tissue realignment of the extensor mechanism. Although positive results were observed after arthroscopic resection of the fibrous nodule [12,16,18,19], Beight et al. [2] reported clunk recurrence at an average 24.3 months in four out of 11 patients who were treated arthroscopically. Three clunks in the study by Beight et al. [2] underwent initial arthrotomy and patellar button revision, with no recurrence. Five patients in the series of six clunks by Ip et al. [3] underwent surgical treatment without recurrence at 29-month follow-up (on average): four patients were treated with arthroscopic debridement, and one patient was treated with open resection of the nodule associated with lateral retinacular release. Takahashi et al. [18] stated that arthroscopic treatment brings good results in typical patellar clunk with the presence of fibrous nodule, while results are less predictable with other types of peripatellar soft-tissue impingement. Extreme caution is recommended to avoid iatrogenic damage to the prosthetic components during arthroscopy [8]. Therefore, open treatment remains a valid therapeutic option that safely allows complete intra-articular synovial debridement, with possible patellar button revision or other additional procedures when needed.

## Conclusions

Despite the excellent clinical results of posterior stabilized total knee replacement, patellar clunk syndrome continues to pose very disturbing problems for both surgeons and patients. Although the syndrome seems to have multifactorial etiology, and encompasses a wide spectrum of pathologic lesions, the use of modern prosthesis along with meticulous surgical technique should greatly reduce its occurrence. When prosthetic components are correctly positioned, arthroscopic or open debridement of the peripatellar fibrous formations usually leads to symptom relief.
